# Supplementary figures and images for: Differentially Expressed Circular RNAs in Peripheral Blood Mononuclear Cells of Patients with Parkinson's Disease
Source: Mov Disord. 2021 Jan 12;36(5):1170–9. doi: 10.1002/mds.28467 (PMC8248110; doi:10.1002/mds.28467)

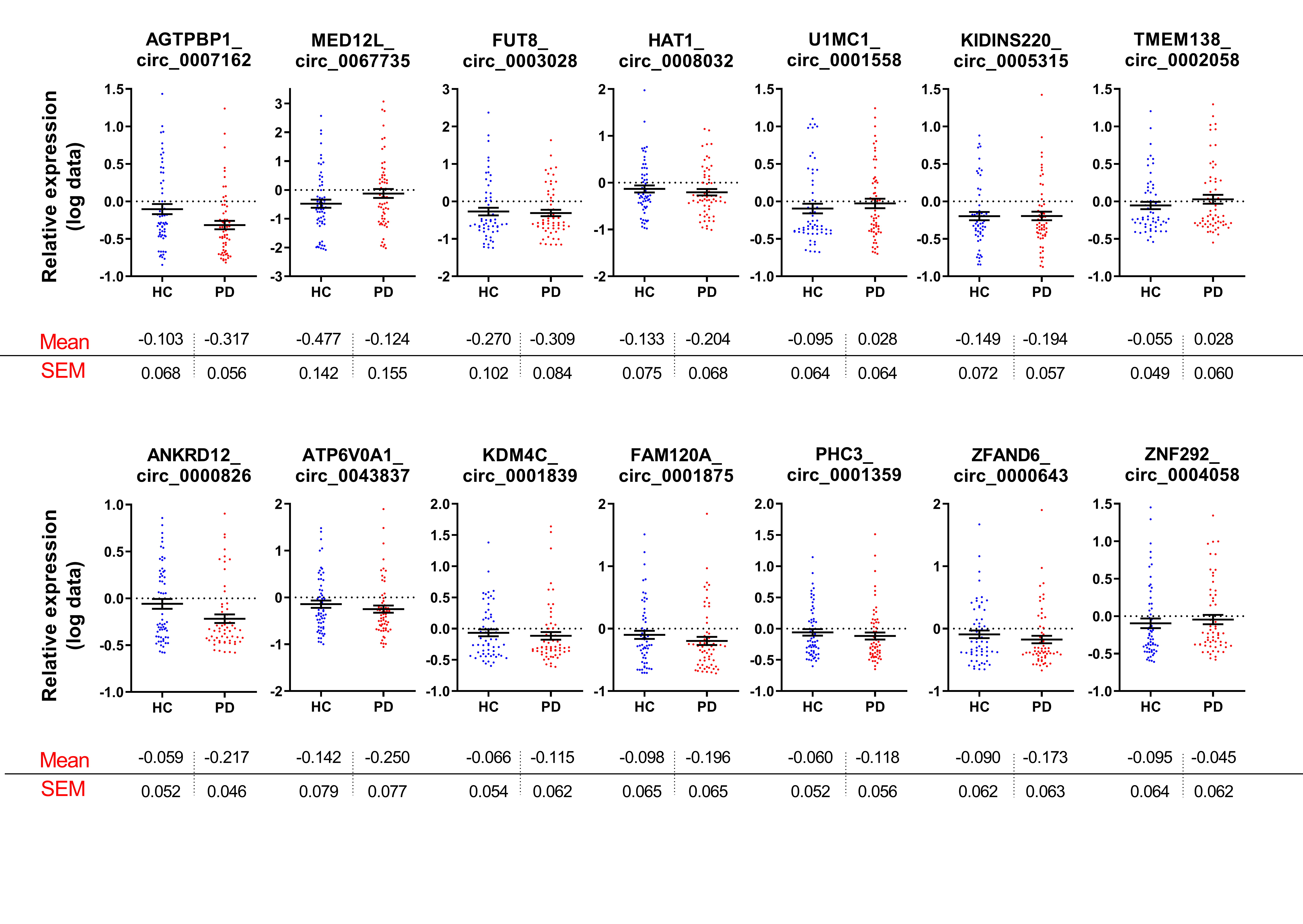

Supplement: Supplementary file 1 — Figure S1. Swarm plots for the 42 circRNAs whose relative expression is not significantly altered in the PBMCs of idiopathic PD patients. Mean levels +/− SEM are included below each graph. Graphs demonstrate relative expression of log‐transformed data. Unpaired t‐test was used to determine the significance of differences between the two groups. *P < 0.05, **P < 0.01, ***P < 0.001. [file MDS-36-1170-s002.zip › MDS_28467_Suppl Fig 1A.tif]

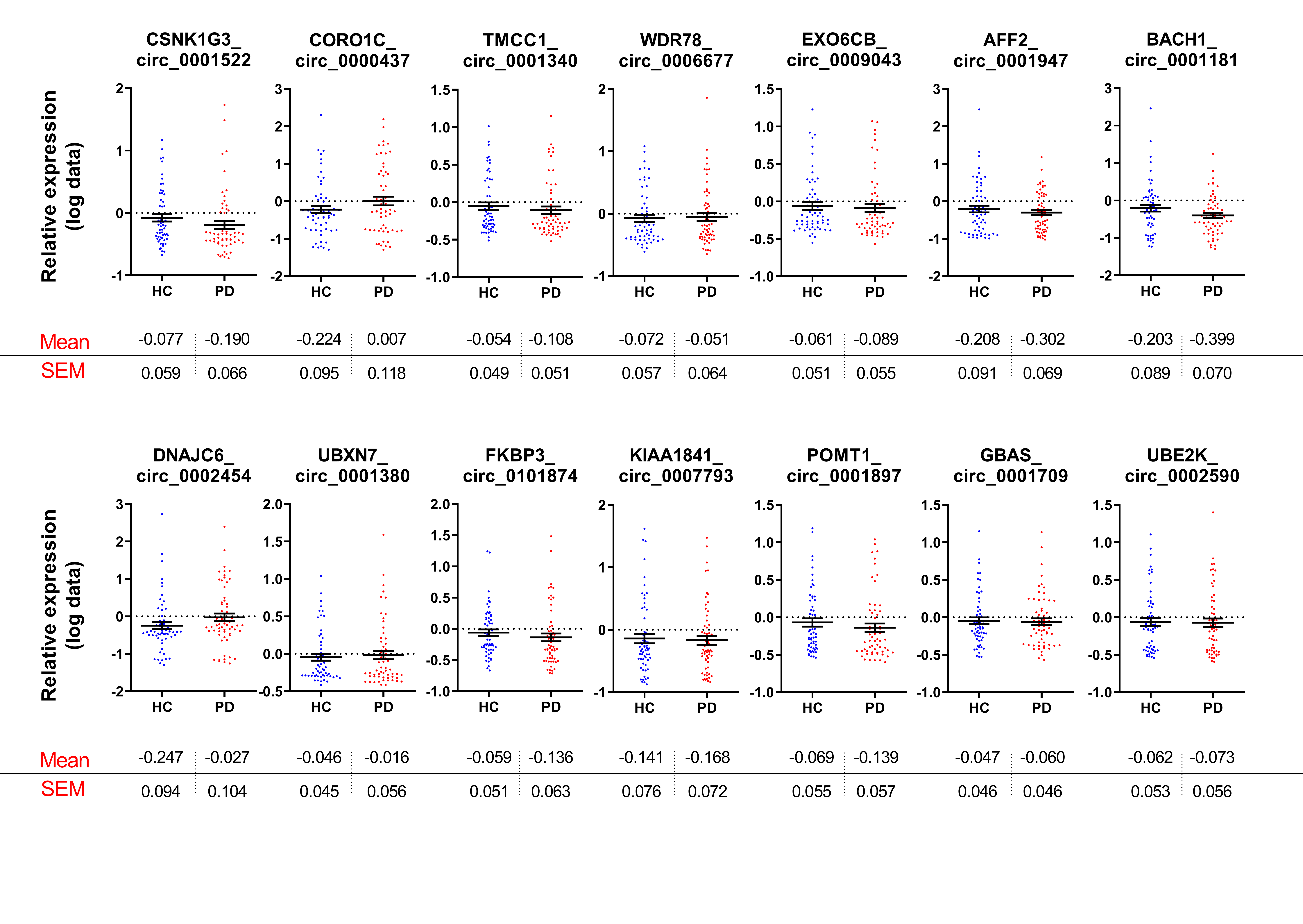

Supplement: Supplementary file 1 — Figure S1. Swarm plots for the 42 circRNAs whose relative expression is not significantly altered in the PBMCs of idiopathic PD patients. Mean levels +/− SEM are included below each graph. Graphs demonstrate relative expression of log‐transformed data. Unpaired t‐test was used to determine the significance of differences between the two groups. *P < 0.05, **P < 0.01, ***P < 0.001. [file MDS-36-1170-s002.zip › MDS_28467_Suppl Fig 1B.tif]

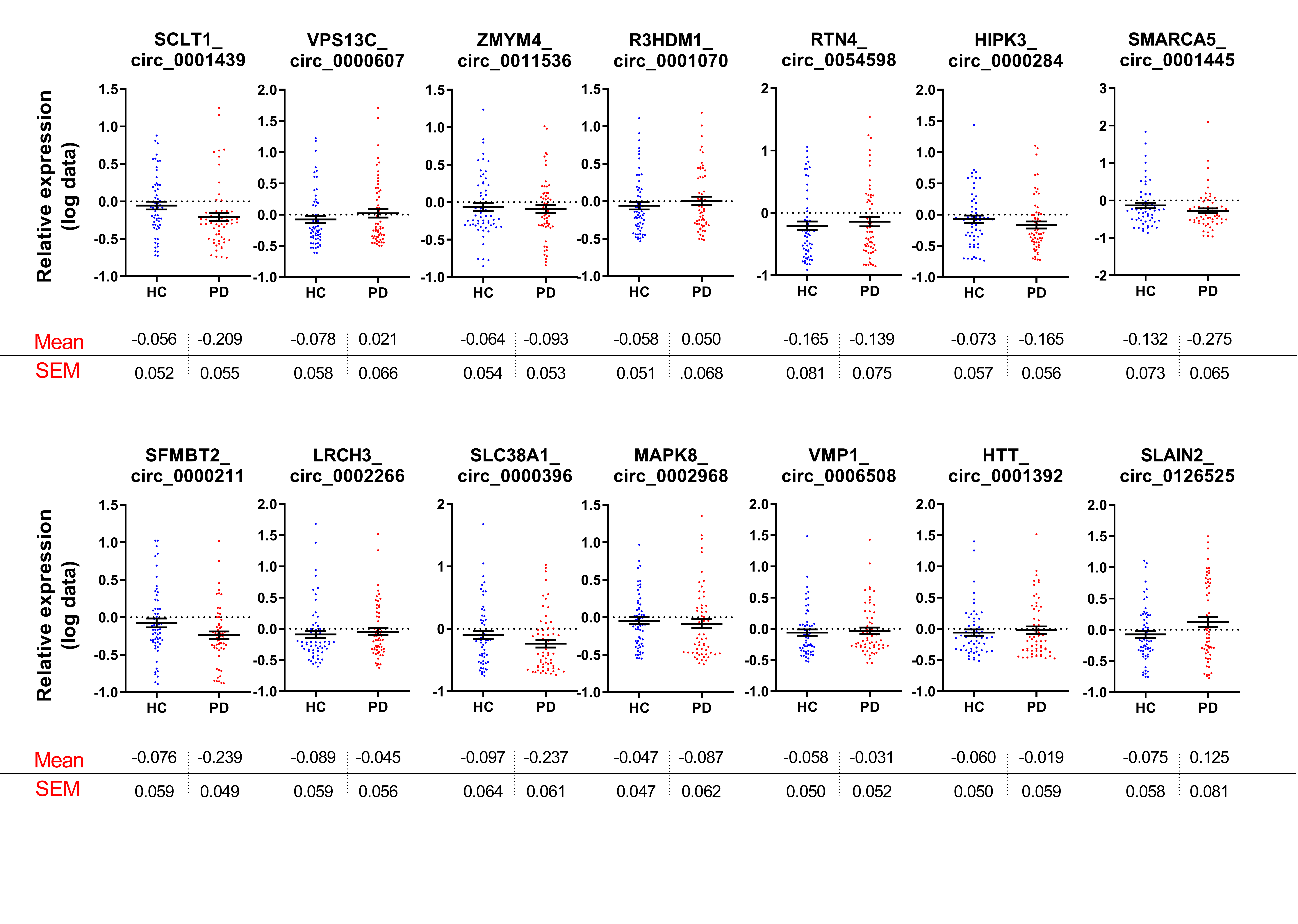

Supplement: Supplementary file 1 — Figure S1. Swarm plots for the 42 circRNAs whose relative expression is not significantly altered in the PBMCs of idiopathic PD patients. Mean levels +/− SEM are included below each graph. Graphs demonstrate relative expression of log‐transformed data. Unpaired t‐test was used to determine the significance of differences between the two groups. *P < 0.05, **P < 0.01, ***P < 0.001. [file MDS-36-1170-s002.zip › MDS_28467_Suppl Fig 1C.tif]

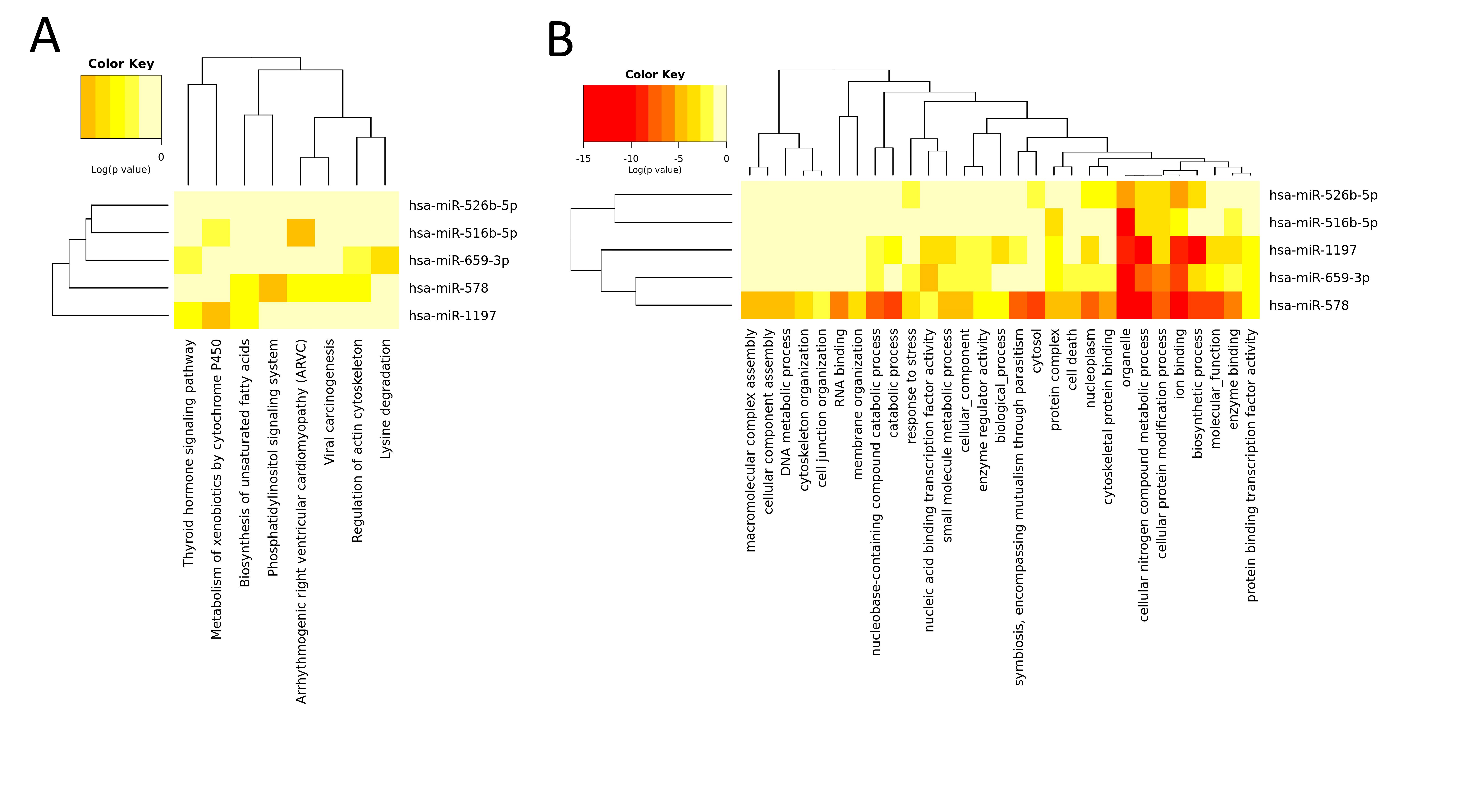

Supplement: Supplementary file 2 — Figure S2. KEGG and GOslim classifications of the circRNA‐miRNA target genes. (A) KEGG and (B) GOslim categories union of the RNA targets of the five miRNAs sequestered by three or more of the deregulated PD circRNAs (miR‐516b‐5p, miR‐526b‐5p, miR‐578, miR‐659‐3p, miR‐1197). They were prepared using the DIANA‐miRPath v3.0 interface using default values (P‐value threshold 0.05, microT‐CDS threshold 0.8). [file MDS-36-1170-s004.tif]
