# Supplementary material for: Differentially Expressed Circular RNAs in Peripheral Blood Mononuclear Cells of Patients with Parkinson's Disease
Source: Mov Disord. 2021 Jan 12;36(5):1170–9. doi: 10.1002/mds.28467 (PMC8248110; doi:10.1002/mds.28467)
Supplement: Supplementary file 3 — Table S1. Primer sequences used for real‐time PCR. [file MDS-36-1170-s001.docx]

**Supplemental Table 1. Primer sequences used for real-time PCR.**

| **circRNA** | **Forward** | **Reverse** |
| --- | --- | --- |
| hsa_AFF2_circ_0001947 | ACTGCCAAAGTTCACCATCC | AGTTTCCAAGCGTGTTCTGG |
| hsa_AGTPBP1_circ_0007162 | GTGGATTCGTATGGGGACTG | CCTGCCGTAGGAACAACAAT |
| hsa_ANKRD12_circ_0000826 | CGTCCAGTGGATGTAGCAGA | AACCCAGATTTGGGCATTTT |
| hsa_ANKS1B_circ_0007294 | TGGGAAAAATGGAAGCCAGAGT | AGAACTCGTACAACATCCACCT |
| hsa_ARPP21_circ_0001281 | GTCCATCGAGTGGCAGCTT | ATGCCCATCTCCATGCGATT |
| hsa_ATP6V0A1 _circ_0043837 | CGCCGTCAGTATTTGAGGAG | GGCTGACTCCAAACAGCATA |
| hsa_BACH1_circ_0001181 | CGCTGTCGCAAGAGAAAAC | AGGCAAAAACCGAGTTCTCA |
| hsa_CDR1_circ_0001946 | CATGTCTTCCAACGTCTCCA | ACCTTGACACAGGTGCCATC |
| hsa_CNTNAP2_circ_0133631 | GGATGCTCTACAGCGACACA | GGGAGTCCAGAGACAAGTGG |
| hsa_CORO1C_circ_0000437 | ATGGGTTACATGCCCAAGAG | ACACCCCATTGCTAGTGTCC |
| hsa_CSNK1G3_circ_0001522 | GCACCACAGCTACATTTGGA | GCATGTTCATCCCATTCGT |
| hsa_DAB1_circ_0113684 | TGTGCCAAAAAGTCAACCTG | CCCGGTGATCTGTAATGTCC |
| hsa_DGKB_circ_0133622 | AGGAGACCCAGTGCCTTACA | GGTCCTTCAAAGGTCCACAG |
| hsa_DNAJC6_circ_0002454 | TGACATTCGAAGCTTTTTGG | ATAGCTGGGCTCCATGTCTG |
| hsa_ DOP1B_circ_0001187 | GGAATGTTCTCAGAAAGGAGGA | AATGTTTTCCCTCTTGGAGGT |
| hsa_ERC2_circ_0124264 | CGGATTGAGAGGAGGAAACA | TTGATTGTGCTTGAGGTTGG |
| hsa_EXOC6B_circ_0009043 | TCCGCAAACATTCAGACAAA | GCTTCAGCTCTTCCATTGCT |
| hsa_EXOSC1_circ_0005887 | CCCGAATTCTTGCAGACCTA | ACCTGGGCGGAAACTCTTAT |
| hsa_FAM120A_circ_0001875 | TGTCATTTCATCCACCACATT | CAAGCCATGGAAACCATTCT |
| hsa_FGD4_circ_0000390 | TAGCTGCTCGGAACACTTCA | GCATGTGATGCTGCAAAGTT |
| hsa_FKBP3_circ_0101874 | AAGGCTCGACTGGAGATTGA | GCTGTCTTGGCCACATTTTT |
| hsa_FUT8_circ_0003028 | GTCCAAGATTCTGGCAAAGC | TCAAAGAGATCCTCCTGGTGA |
| hsa_GBAS_circ_0001709 | GAATGAGCCTGTGCCAAGAT | TTCTGTGAGGGCTGGATAGC |
| hsa_GRIN2B_circ_0097968 | CTGAGCCCAAAAGCAGTTGT | TTACGGAAGCTTGCTGTTCA |
| hsa_HAGH_circ_0105101 | CAGGGAGAAGGACCAGTTCA | GCTCCCCGATGCTGTACTT |
| hsa_HAT1_circ_0008032 | TGCTGGTAGCCTGTCAACAA | GCCAGTTTCTTCTCCACTGC |
| hsa_HIPK3_circ_0000284 | TCGGCCAGTCATGTATCAAA | GGGTAGACCAAGACTTGTGAGG |
| hsa_HMGCLL1_circ_0131944 | CAGGTGCTCTTGCTGTTCAC | CATCCCTAGGCCCAACTTCT |
| hsa_HOMER1_circ_0006916 | AATGCATTGCCATTTTCACA | TGTGTTTGGGTCAATTTGGA |
| hsa_HTT_circ_0001392 | TTTGGCAATTTTGCAAATGA | CACACGGTCTTTCTTGGTAGC |
| hsa_KCNN2_circ_0127664 | CCGAGCTTGTGAAAGTTGTTC | CACACACCAGTATTTCCAAGCA |
| hsa_KDM4C_circ_0001839 | ATGGCTACCATGCTGGTTTT | TCAAGTCGTTTTCCATGCTC |
| hsa_KIAA1841_circ_0007793 | GGACCGAGTCAAGTCAAAGG | TGGGCACAGGGTGAAGATAC |
| hsa_KIDINS220_circ_0005315 | ACCGGACTTCATGGCTCATA | CCTCTGCATCTGCCTTCTTC |
| hsa_KLHL1_circ_0100796 | GGAATTCGAGCCTTCGCAGA | TGGAGTCCAAATCACTTTGAGGT |
| hsa_LMBR1_circ_0005939 | GGCTAAATGGCTCCCTGATT | CGATGGCATCTTCATCTTCTT |
| hsa_LPAR1_circ_0087960 | GGCTGCCATCTCTACTTCCA | GTGGATGGGGAGCTTCATAA |
| hsa_LRCH1_circ_0002215 | GGAAGCATGCCGAAAATTAG | TCCTCCGGATTGTAAACTGC |
| hsa_LRCH3_circ_0002266 | TCCCAAATTGGTAACCTGGA | CCAGAGAAACAAAGTGACATGCT |
| hsa_LRRC7_circ_0114013 | TGGACAAGGAAATCCATTCA | TCCATTGGCCACTAGGAGAT |
| hsa_MAPK8_circ_0002968 | TGTGGAATCAAGCACCTTCA | AAAAATTGTTGTCACGCTTGC |
| hsa_MAPK9_circ_0001566 | TGGAGCTGGATCATGAAAGA | AAGGGTGGGCAAGTTTCAG |
| hsa_MED12L_circ_0067735 | CAACGTGGCTGATCAAGATG | GGCTGGCTGATTATTGAAGC |
| hsa_NRXN1_circ_0054525 | TTCCAGGGTCACCAGTCAGT | GATCCTTTGAACGTGGCAAT |
| hsa_NTRK2_circ_0139142 | CCTGAATGAAAGCAGCAAGA | ACCTTTTCTGGTTTGCGATG |
| hsa_PAK3_circ_0139566 | GTTGGGGACCCAAAGAAAAA | ATCAAACCCCACATGAATCG |
| hsa_PDE4B_circ_0008433 | ACTGCCTTTGACAACGCTTC | TTCAATGCAGTTTGCTGACA |
| hsa_PHC3_circ_0001359 | TGTCACCCGGACATCAAGTA | GGGTAATACTGCCGCTGGTA |
| hsa_POMT1_circ_0001897 | CTGGCCTTGGGAGGTTATTT | GCCCACTGTCATCCAAGAAG |
| hsa_PPP2R2B_circ_0128256 | GCGTGATAAGAGGCCAGAAG | AATTCTCCCGTGTGGTTGAA |
| hsa_PRKCB_circ_0000682 | ACAGGGACGTCCTCATTGTC | GTCACATTTCATCCCCTGGT |
| hsa_PSD3_circ_0002111 | TGCAAGGGGTAAATGAGGGTG | TTCAGTGCTCCCCATTTCAGA |
| hsa_PSEN1_circ_0003848 | AGTTACCTGCACCGTTGTCC | GCTGTCTAAGGACCGCAAAG |
| hsa_PTK2_circ_0003171 | GATGGCTCCAGAGTCAATCAA | CTCACGCTGTCCGAAGTACA |
| hsa_R3HDM1_circ_0001070 | AAGGATCTATGCCCACAACAGG | GACTCTGGGGTTGCTGAACT |
| hsa_REPS1_circ_0004368 | CCATTCAGCCTGATCTAAACG | ACACTGCATCACCAGCAGAA |
| hsa_RERE_circ_0002158 | TAAAGCCCGAGTGGATTCAT | TGACGTTCATGAGGAGATGG |
| hsa_RIMS1_circ_0132246 | GGAGATCCAGCCTTAGTGCC | AGCTTGCTTTTGTGGAAGAGT |
| hsa_RIMS1_ circ_0132250 | AGCAGGTGGAAAGAAACGGA | TGTCTTGTTCGCTCCTCCAC |
| hsa_RIMS2_circ_0005114 | TCTGTCACGGAAAAGTCGCA | TGATCCGGCTACCTGTTTGT |
| hsa_RMST_circ_0099634 | AGGGGCTAGTTGAGGAATGG | CCATTCTGTGCTGAGTGGAG |
| hsa_RTN4_circ_0054598 | GAGGACAGATCACCATCTGCTA | TGCAGAGGAGCGTATCACAG |
| hsa_SCAF8_circ_0001654 | CTTGCGGCTGTAGCTCAGAT | TTTGGGTCATTTTCGCTTTC |
| hsa_SCLT1_circ_0001439 | GAACATGGAAGTGACTAACCAACA | GGGAAAGGCCTCCAATTTT |
| hsa_SFMBT2_circ_0000211 | ATGGCCTCTGAATGGAAATG | CTCGAACCAGTCAAGTCACG |
| hsa_SLAIN1_circ_0000497 | GCTTGCATGGAGCTGGAAATG | ATGTCTACACCACTGCAAAGGA |
| hsa_SLAIN2_circ_0126525 | AAGTGCCAAACGGAGGAATA | AATCCAAAACTTGCCTGCAC |
| hsa_SLC30A6_circ_0005695 | CCGTGCCTGAGCAATAGTTT | TGCACCACATAAGCAGGAAG |
| hsa_SLC38A1_circ_0000396 | CATTATGGGCAGTGGGATTT | GTATGGCCTTCCAGGGTTTT |
| hsa_SMARCA5_circ_0001445 | TGGGCGAAAGTTCACTTAGAA | GAAGCATTTCATCTTTCCCAAT |
| hsa_STX6_circ_0007905 | GGCTGGACAATGTGATGAAG | GCCTGCACAGATGAAGTTGA |
| hsa_SYT1_circ_0099287 | ACAGTGGATTTTGGCCATGT | AAAAGCAGCAGGTCAGGACT |
| hsa_TMCC1_circ_0001340 | GGGAAGCTATTGCTGGGATT | ACCAAAGGCAACTGTTCACC |
| hsa_TMEFF1_circ_0004425 | GGTCAGGGGCAGAAGTTCAC | CTCTTACGTCAGACTCCCTCAC |
| hsa_TMEM132D_circ_0097876 | CAAAGACCGTGAGGAAAGGA | GCAAGGAAAACCTCTGGATG |
| hsa_TMEM138_circ_0002058 | GCTGGAAAAACTCCAACAGC | AGCCTGGAAGACGAAGGTGT |
| hsa_UBE2K_circ_0002590 | CAGAGCCAGATGATCCACAG | GTGTGTCTGGAGGTCCTGCT |
| hsa_UBXN7_circ_0001380 | TGCATAAAGGCAGCTTTGAA | CCGTCGTCTTTTAGGAGCAC |
| hsa_UIMC1_circ_0001558 | CCCACAAAGATTGAACGACA | GCAGCAACACTTTGTGAAGC |
| hsa_UNC13C_circ_0103896 | AAAGCAAATGGCAGAGTTGG | AAACCAGAAGCAAAGCTCCA |
| hsa_VMP1_circ_0006508 | TCTTCTGTTGGGCTTGGAAC | TTCCATTCTCTGCCATTTCA |
| hsa_VPS13C_circ_0000607 | AAGCACAGGCAGTTACTCAAGA | GCATGGAGTCCAGTGTTCATT |
| hsa_WDR78_circ_0006677 | GAAGACCTGGAAGAACCATCC | TCTGCTTTGATTTGCACCAG |
| hsa_YY1AP1_circ_0014606 | ATTCTGGACCCAGCACAAAG | TCTTCTGGGCCATCATCTTC |
| hsa_ZFAND6_circ_0000643 | TGTGGACAAAGCAGTACCTGA | CCATTTGTACGAGGGTTTCC |
| hsa_ZMYM4_circ_0011536 | GCACCACAGCAGGGACTACT | ATCCTGTTTGATTCGGCATT |
| hsa_ZNF292_circ_0004058 | TTCTTTCCCAGGAACCATTG | CGGGCTTTAACATAACTTTGG |
| hsa-GAPDH | GCACCACCAACTGCTTAG | GCCATCCACAGTCTTCTG |
| hsa-U6 | CGCTTCGGCAGCACATATAC | TTCACGAATTTGCGTGTCAT |
