# Supplementary material for: Differentially Expressed Circular RNAs in Peripheral Blood Mononuclear Cells of Patients with Parkinson's Disease
Source: Mov Disord. 2021 Jan 12;36(5):1170–9. doi: 10.1002/mds.28467 (PMC8248110; doi:10.1002/mds.28467)
Supplement: Supplementary file 4 — Table S2. List of 39 circRNAs not detected in PBMCs. [file MDS-36-1170-s007.docx]

**Supplemental Table 2.** List of 39 circRNAs not detected in PBMCs

| ANKS1B_circ_0007294 | KLHL1_circ_0100796 | RERE_circ_0002158 |
| --- | --- | --- |
| ARPP21_circ_0001281 | LMBR1_circ_0005939 | RIMS1_circ_0132246 |
| CDR1_circ_0001946 | LPAR1_circ_0087960 | RIMS1_circ_0132250 |
| CNTNAP2_circ_0133631 | LRCH1_circ_0002215 | [RIMS2_circ_0005114](http://www.circbase.org/cgi-bin/singlerecord.cgi?id=hsa_circ_0005114) |
| DAB1_circ_0113684 | LRRC7_circ_0114013 | RMST_circ_0099634 |
| DGKB_circ_0133622 | NRXN1_circ_0054525 | SCAF8_circ_0001654 |
| ERC2_circ_0124264 | NTRK2_circ_0139142 | SLC30A6_circ_0005695 |
| EXOSC1_circ_0005887 | PAK3_circ_0139566 | STX6_circ_0007905 |
| FGD4_circ_0000390 | PDE4B_circ_0008433 | SYT1_circ_0099287 |
| GRIN2B_circ_0097968 | PPP2R2B_circ_0128256 | TMEFF1_circ_0004425 |
| HAGH_circ_0105101 | PRKCB_circ_0000682 | TMEM132D_circ_0097876 |
| HMGCLL1_circ_0131944 | PSD3_circ_0002111 | UNC13C_circ_0103896 |
| KCNN2_circ_0127664 | PTK2_circ_0003171 | YY1AP1_circ_0014606 |
