# Supplementary material for: Differentially Expressed Circular RNAs in Peripheral Blood Mononuclear Cells of Patients with Parkinson's Disease
Source: Mov Disord. 2021 Jan 12;36(5):1170–9. doi: 10.1002/mds.28467 (PMC8248110; doi:10.1002/mds.28467)
Supplement: Supplementary file 5 — Table S3. Basic characteristics and comparative PBMC circRNA expression in idiopathic PD patients and healthy controls. CircRNA size and expression in the brain as well as host transcript expression in the body. Means with their respective standard deviation (std) for both groups are shown. Statistically significant differences in comparison to healthy controls (unpaired t‐test) are highlighted in grey. Multiple comparison analysis (adjusted P‐values) calculated according to Benjamini‐Hochberg false discovery rate method. *According to Rybak et al. ** According to genotype‐tissue expression portal. [file MDS-36-1170-s006.docx]

**Supplemental Table 3. Basic characteristics and comparative PBMC circRNA expression in idiopathic PD patients and healthy controls.**

CircRNA size and expression in the brain as well as host transcript expression in the body. Means with their respective standard deviation (std) for both groups are shown. Statistically significant differences in comparison to healthy controls (unpaired t-test) are highlighted in grey. Multiple comparison analysis (adjusted *P* values) calculated according to Benjamini-Hochberg false discovery rate method.

| **circRNA ID** | **Spliced**  **Seq length** | **Expression score in the human brain*** | **Host gene**  **symbol** | **Host gene**  **mRNA expression**  **in the human body**** | **Mean (± std)** | | **Unpaired t-test**  ***P* value** | **Adjusted**  ***P* values** |
| --- | --- | --- | --- | --- | --- | --- | --- | --- |
|  |  |  |  |  | **HC** | **iPD** |  |  |
| circ_0001380 | 247 | 15326 | UBXN7 | Ubiquitous, expressed least in the brain except cerebellum where expression is similar to other tissues | -0.046 (0.348) | -0.016 (0.432) | 0.678 | 0.812 |
| circ_0000284 | 1099 | 13889 | HIPK3 | Ubiquitous, expressed least in the brain | -0.073 (0.443) | -0.165 (0.435) | 0.252 | 0.603 |
| circ_0001445 | 269 | 9354 | SMARCA5 | Ubiquitous, lower expression in the brain | -0.132 (0.565) | -0.275 (0.504) | 0.147 | 0.415 |
| circ_0009043 | 390 | 9121 | EXOC6B | Ubiquitous, enriched in the skin | -0.061 (0.395) | -0.089 (0.425) | 0.701 | 0.812 |
| circ_0001947 | 861 | 9058 | AFF2 | Ubiquitous, highly enriched in the cerebellum | -0.208 (0.702) | -0.302 (0.531) | 0.412 | 0.706 |
| circ_0002590 | 336 | 8841 | UBE2K | Ubiquitous, enriched in the cerebellum | -0.062 (0.409) | -0.073 (0.437) | 0.895 | 0.895 |
| circ_0000437 | 251 | 8402 | CORO1C | Ubiquitous, expressed least in the brain | -0.224 (0.736) | 0.007 (0.911) | 0.130 | 0.390 |
| circ_0000497 | 788 | 6004 | SLAIN1 | Brain-specific | -0.096 (0.520) | -0.447 (0.313) | <0.0001  *** | 0.001*** |
| circ_0054598 | 2457 | 5985 | RTN4 | Ubiquitous, enriched in the brain | -0.165 (0.624) | -0.139 (0.583) | 0.815 | 0.850 |
| circ_0001522 | 536 | 5649 | CSNK1G3 | Ubiquitous, lower expression in the brain | -0.077 (0.452) | -0.190 (0.505) | 0.205 | 0.518 |
| circ_0001070 | 307 | 5300 | R3HDM1 | Brain-specific | -0.058 (0.394) | 0.050 (0.523) | 0.204 | 0.518 |
| circ_0011536 | 755 | 4662 | ZMYM4 | Ubiquitous, expressed least in the brain except cerebellum where expression is somewhat higher than in other tissues | -0.064 (0.417) | -0.093 (0.406) | 0.694 | 0.812 |
| circ_0000607 | 606 | 4056 | VPS13C | Ubiquitous, expressed least in the brain except cerebellum where expression is similar to other tissues | -0.078 (0.449) | 0.021 (0.514) | 0.266 | 0.603 |
| circ_0001439 | 396 | 3870 | SCLT1 | Ubiquitous, expressed least in the brain except cerebellum where expression is somewhat higher than in other tissues | -0.056 (0.399) | -0.209 (0.429) | 0.046* | 0.210 |
| circ_0000826 | 994 | 3868 | ANKRD12 | Ubiquitous, expressed least in the brain except cerebellum where expression is somewhat higher than in other tissues | -0.059 (0.405) | -0.217 (0.355) | 0.024* | 0.142 |
| circ_0000211 | 434 | 3783 | SFMBT2 | Ubiquitous | -0.076 (0.457) | -0.239 (0.381) | 0.036* | 0.189 |
| circ_0002266 | 378 | 3204 | LRCH3 | Ubiquitous, expressed least in the brain except cerebellum where expression is somewhat higher than in other tissues | -0.089 (0.458) | -0.045 (0.433) | 0.595 | 0.782 |
| circ_0002968 | 499 | 3123 | MAPK8 | Ubiquitous, highly enriched in the cerebellum | -0.047 (0.361) | -0.087 (0.476) | 0.603 | 0.782 |
| circ_0000396 | 522 | 3086 | SLC38A1 | Ubiquitous | -0.097 (0.498) | -0.237 (0.472) | 0.115 | 0.380 |
| circ_0001181 | 1836 | 3082 | BACH1 | ubiquitous, expressed least in the brain | -0.203 (0.691) | -0.399 (0.538) | 0.086 | 0.344 |
| circ_0006916 | 522 | 3026 | HOMER1 | Ubiquitous, enriched in the brain | -0.076 (0.452) | -0.292 (0.316) | 0.003** | 0.036* |
| circ_0001709 | 212 | 2889 | GBAS | Ubiquitous, highly enriched in the skeletal muscle | -0.047 (0.354) | -0.060 (0.359) | 0.839 | 0.857 |
| circ_0001897 | 158 | 2882 | POMT1 | Ubiquitous, highly enriched in the cerebellum and testis | -0.069 (0.426) | -0.139 (0.439) | 0.375 | 0.667 |
| circ_0101874 | 502 | 2687 | FKBP3 | Ubiquitous, enriched in the brain | -0.059 (0.395) | -0.136 (0.482) | 0.347 | 0.640 |
| circ_0002454 | 350 | 2511 | DNAJC6 | Brain-specific | -0.247 (0.728) | -0.027 (0.806) | 0.119 | 0.380 |
| circ_0001558 | 397 | 2476 | UIMC1 | Ubiquitous, expressed least in the brain except cerebellum where expression is similar to other tissues | -0.095 (0.498) | 0.028 (0.497) | 0.459 | 0.711 |
| circ_0001187 | 301 | 2263 | DOP1B | Ubiquitous, highly enriched in the cerebellum | -0.071 (0.440) | -0.265 (0.282) | 0.005** | 0.038* |
| circ_0006508 | 440 | 2229 | VMP1 | Ubiquitous, expressed least in the brain | -0.058 (0.384) | -0.031 (0.402) | 0.711 | 0.812 |
| circ_0006677 | 473 | 2190 | WDR78 | Testis, lung | -0.072 (0.439) | -0.051 (0.492) | 0.801 | 0.850 |
| circ_0008032 | 302 | 2153 | HAT1 | Ubiquitous, expressed least in the brain | -0.133 (0.579) | -0.204 (0.526) | 0.482 | 0.723 |
| circ_0043837 | 444 | 1892 | ATP6V0A1 | Ubiquitous, highly enriched in the brain | -0.142 (0.611) | -0.250 (0.597) | 0.334 | 0.640 |
| circ_0004368 | 352 | 1882 | REPS1 | Ubiquitous, highly enriched in the cerebellum | -0.076 (0.455) | -0.299 (0.313) | 0.002** | 0.036* |
| circ_0005315 | 308 | 1820 | KIDINS220 | Ubiquitous, enriched in the cerebellum | -0.149 (0.554) | -0.194 (0.438) | 0.628 | 0.793 |
| circ_0002058 | 248 | 1775 | TMEM138 | ubiquitous, expressed least in the brain | -0.055 (0.379) | 0.028 (0.465) | 0.286 | 0.603 |
| circ_0001566 | 497 | 1745 | MAPK9 | Ubiquitous, enriched in the brain | -0.066 (0.425) | -0.284 (0.352) | 0.002** | 0.036* |
| circ_0007793 | 284 | 1645 | KIAA1841 | Ubiquitous, somewhat brain-enriched | -0.141 (0.591) | -0.168 (0.556) | 0.793 | 0.850 |
| circ_0001340 | 251 | 1631 | TMCC1 | Ubiquitous, enriched in the cerebellum | -0.054 (0.380) | -0.108 (0.389) | 0.443 | 0.711 |
| circ_0007162 | 1121 | 1526 | AGTPBP1 | Ubiquitous, somewhat brain-enriched | -0.103 (0.524) | -0.317 (0.434) | 0.017* | 0.113 |
| circ_0003848 | 222 | 1430 | PSEN1 | Ubiquitous, highly enriched in the spinal cord | -0.054 (0.388) | -0.241 (0.308) | 0.004** | 0.038* |
| circ_0004058 | 370 | 1422 | ZNF292 | Ubiquitous, expressed least in the brain except cerebellum where expression is similar to other tissues | -0.095 (0.491) | -0.045 (0.479) | 0.577 | 0.782 |
| circ_0001875 | 556 | 1345 | FAM120A | Ubiquitous, expressed least in the brain | -0.098 (0.504) | -0.196 (0.500) | 0.289 | 0.603 |
| circ_0003028 | 430 | 1336 | FUT8 | Ubiquitous, highly enriched in the spinal cord | -0.270 (0.792) | -0.309 (0.654) | 0.850 | 0.850 |
| circ_0001839 | 292 | 1280 | KDM4C | Ubiquitous, highly enriched in the cerebellum | -0.066 (0.418) | -0.115 (0.482) | 0.557 | 0.782 |
| circ_0001392 | 484 | 1270 | HTT | Ubiquitous, enriched in the cerebellum | -0.060 (0.384) | -0.019 (0.453) | 0.602 | 0.782 |
| circ_0067735 | 457 | 952 | MED12L | Adrenal glands, brain, cervix, pituitary and testis | -0.477 (1.100) | -0.124 (1.202) | 0.096 | 0.354 |
| circ_0000643 | 381 | 761 | ZFAND6 | Ubiquitous, expressed least in the brain | -0.090 (0.477) | -0.173 (0.467) | 0.341 | 0.640 |
| circ_0126525 | 971 | 720 | SLAIN2 | Ubiquitous, expressed least in the brain | -0.075 (0.450) | 0.125 (0.628) | 0.048* | 0.210 |
| circ_0001359 | 505 | 709 | PHC3 | Ubiquitous, expressed least in the brain | -0.060 (0.403) | -0.118 (0.434) | 0.454 | 0.711 |

*According to Rybak et al.

** According to GTEx portal.
