# Supplementary material for: Differentially Expressed Circular RNAs in Peripheral Blood Mononuclear Cells of Patients with Parkinson's Disease
Source: Mov Disord. 2021 Jan 12;36(5):1170–9. doi: 10.1002/mds.28467 (PMC8248110; doi:10.1002/mds.28467)
Supplement: Supplementary file 6 — Table S4. Correlation between relative circRNA expression and sex, age, age‐at‐onset, PD duration, HY, UPDRS and MMSE scores and LEDD of PD patients. Significance level is set at P < 0.0005 after Bonferroni correction. [file MDS-36-1170-s005.docx]

**Supplemental Table 4. Correlation between relative circRNA expression and sex, age, age-at-onset, PD duration, HY, UPDRS and MMSE scores and LEDD of PD patients.**

| **circRNA** | **Sex**  **U score**  **(*P* value)** | **Age**  **Rho**  **(*P* value)** | **Age-at-onset**  **Rho**  **(*P* value)** | **PD duration**  **Rho**  **(*P* value)** | **UPDRS III**  **Rho**  **(*P* value)** | **MMSE**  **Rho**  **(*P* value)** | **HY**  **Rho**  **(*P* value)** | **LEDD**  **Rho**  **(*P* value)** |
| --- | --- | --- | --- | --- | --- | --- | --- | --- |
| UBXN7_circ  _0001380 | 0.027  (0.8684) | -0.012  (0.8949) | -0.142  (0.2831) | 0.041  (0.7579) | 0.143  (0.2788) | 0.096  (0.4674) | 0.113  (0.3953) | 0.055  (0.6791) |
| HIPK3_circ  _0000284 | 1.694  (0.1930) | 0.004  (0.9627) | -0.129  (0.3266) | -0.063  (0.6303) | -0.036  (0.7847) | 0.090  (0.4924) | -0.052  (0.6943) | 0.100  (0.4450) |
| SΜARCA5_  circ_0001445 | 0.778  (0.3776) | -0.035  (0.7078) | -0.119  (0.3663) | -0.174  (0.1847) | 0.018  (0.8931) | 0.082  (0.5321) | 0.005  (0.9704) | 0.006  (0.9643) |
| EXO6CB_circ_0009043 | 1.129  (0.2880) | 0.142  (0.1215) | 0.225  (0.0836) | -0.116  (0.3793) | -0.014  (0.9165) | -0.021  (0.8736) | -0.066  (0.6187) | -0.024  (0.8551) |
| AFF2_circ  _0001947 | 0.011  (0.9159) | 0.107  (0.2446) | 0.101  (0.4418) | -0.036  (0.7871) | 0.050  (0.7055) | -0.048  (0.7130) | -0.138  (0.2917) | 0.156  (0.2334) |
| UBE2K_circ  _0002590 | 0.010  (0.9208) | -0.035  (0.7022) | -0.143  (0.2773) | -0.187  (0.1535) | -0.167  (0.2024) | 0.065  (0.6205) | -0.012  (0.9295) | -0.139  (0.2898) |
| CORO1C_circ_0000437 | 0.035  (0.8521) | -0.089  (0.3313) | -0.390  (0.0021) | 0.211  (0.1050) | 0.090  (0.4928) | 0.039  (0.7691) | 0.019  (0.8856) | 0.183  (0.1618) |
| SLAIN1_circ  _0000497 | 0.026  (0.8717) | -0.098  (0.2870) | -0.085  (0.5188) | -0.109  (0.4071) | 0.094  (0.4771) | 0.257  (0.0472) | 0.132  (0.3146) | 0.050  (0.7061) |
| RTN4_circ  _0054598 | 3.005  (0.0830) | 0.035  (0.7015) | -0.203  (0.1191) | -0.073  (0.5807) | -0.033  (0.8049) | 0.077  (0.5578) | -0.016  (0.9048) | 0.027  (0.8353) |
| CSNK1G3_  circ_0001522 | 3.347  (0.0673) | -0.049  (0.5978) | 0.083  (0.5300) | -0.217  (0.0987) | -0.018  (0.8931) | 0.172  (0.1924) | -0.088  (0.5066) | -0.115  (0.3837) |
| R3HDM1_  circ_0001070 | 0.078  (0.7798) | 0.010  (0.9160) | 0.033  (0.8001) | -0.127  (0.3322) | -0.224  (0.0850) | 0.061  (0.6445) | -0.156  (0.2326) | -0.153  (0.2434) |
| ZMYM4_circ_0011536 | 3.556  (0.0593) | -0.085  (0.3584) | -0.044  (0.7413) | -0.341  (0.0078) | -0.123  (0.3479) | 0.122  (0.3540) | 0.000  (0.9976) | -0.081  (0.5403) |
| VPS13C_circ_0000607 | 0.001  (0.9802) | 0.049  (0.5951) | 0.018  (0.8923) | -0.048  (0.7134) | -0.073  (0.5790) | -0.204  (0.1181) | 0.090  (0.4930) | -0.058  (0.6610) |
| SCLT1_circ  _0001439 | 0.028  (0.8668) | 0.016  (0.8636) | -0.159  (0.2264) | -0.037  (0.7774) | 0.094  (0.4769) | 0.048  (0.7169) | 0.055  (0.6761) | 0.184  (0.1589) |
| ANKRD12_  circ_0000826 | 0.285  (0.5931) | 0.008  (0.9346) | -0.091  (0.4876) | 0.016  (0.9014) | 0.090  (0.4944) | -0.023  (0.8612) | 0.165  (0.2071) | 0.116  (0.3755) |
| SFMBT2_circ_0000211 | 0.519  (0.4711) | 0.100  (0.2778) | 0.008  (0.9543) | -0.114  (0.3873) | 0.053  (0.6898) | 0.244  (0.0598) | 0.092  (0.4864) | 0.003  (0.9828) |
| LRCH3_circ  _0002266 | 0.556  (0.4559) | -0.042  (0.6471) | -0.094  (0.4739) | -0.236  (0.0692) | -0.104  (0.4283) | 0.087  (0.5074) | 0.070  (0.5965) | -0.061  (0.6453) |
| MAPK8_circ  _0002968 | 0.846  (0.3578) | -0.064  (0.4898) | 0.037  (0.7785) | -0.163  (0.2146) | 0.040  (0.7634) | 0.096  (0.4648) | 0.115  (0.3814) | -0.090  (0.4943) |
| SLC38A1_  circ_0000396 | 0.062  (0.8037) | -0.111  (0.2284) | 0.207  (0.1124) | -0.149  (0.2565) | 0.024  (0.8558) | 0.087  (0.5068) | 0.004  (0.9766) | -0.135  (0.3040) |
| BACH1_circ  _0001181 | 3.978  (0.0461) | -0.133  (0.1488) | -0.110  (0.4034) | -0.028  (0.8333) | 0.070  (0.5948) | 0.070  (0.5932) | 0.066  (0.6146) | 0.254  (0.0498) |
| HOMER1_  circ_0006916 | 0.102  (0.7490) | -0.112  (0.2253) | -0.040  (0.7623) | -0.090  (0.4928) | 0.099  (0.4499) | 0.068  (0.6068) | 0.157  (0.2318) | 0.032  (0.8105) |
| GBAS_circ  _0001709 | 0.892  (0.3450) | -0.041  (0.6604) | -0.066  (0.6138) | -0.001  (0.9951) | 0.111  (0.4003) | 0.201  (0.1244) | 0.185  (0.1566) | 0.025  (0.8487) |
| POMT1_circ  _0001897 | 1.293  (0.2555) | 0.032  (0.7285) | 0.002  (0.9851) | -0.130  (0.3239) | -0.006  (0.9630) | -0.095  (0.4723) | 0.012  (0.9301) | -0.011  (0.9336) |
| FKBP3_circ  _0101874 | 0.030  (0.8633) | -0.073  (0.4314) | -0.009  (0.9472) | -0.142  (0.2839) | 0.104  (0.4318) | 0.111  (0.4040) | 0.016  (0.9041) | 0.050  (0.7091) |
| DNAJC6_circ_0002454 | 0.575  (0.4485) | -0.107  (0.2443) | -0.379  (0.0028) | 0.000  (0.9985) | -0.011  (0.9352) | 0.019  (0.8864) | -0.008  (0.9514) | 0.078  (0.5516) |
| U1MC1_circ  _0001558 | 2.384  (0.1226) | -0.010  (0.9113) | -0.070  (0.5960) | -0.223  (0.0868) | -0.181  (0.1657) | 0.083  (0.5273) | -0.104  (0.4310) | -0.035  (0.7909) |
| DOP1B_circ  _0001187 | 0.812  (0.3676) | -0.035  (0.7063) | 0.020  (0.8778) | -0.101  (0.4416) | -0.147  (0.2623) | -0.075  (0.5711) | -0.156  (0.2354) | -0.267  (0.0396) |
| VMP1_circ  _0006508 | 0.785  (0.3756) | -0.071  (0.4460) | -0.188  (0.1534) | -0.123  (0.3538) | -0.053  (0.6923) | 0.166  (0.2081) | 0.046  (0.7298) | -0.112  (0.3980) |
| WDR78_circ  _0006677 | 0.547  (0.4596) | -0.086  (0.3541) | -0.252  (0.0545) | -0.074  (0.5794) | -0.037  (0.7812) | 0.071  (0.5919) | 0.044  (0.7407) | 0.001  (0.9918) |
| HAT1_circ  _0008032 | 4.783  (0.0287) | -0.039  (0.6710) | -0.098  (0.4550) | -0.131  (0.3176) | 0.018  (0.8914) | 0.100  (0.4488) | 0.138  (0.2922) | 0.030  (0.8196) |
| ATP6V0A1_  circ_0043837 | 0.010  (0.9208) | 0.048  (0.6025) | -0.111  (0.3978) | 0.095  (0.4719) | -0.001  (0.9958) | -0.008  (0.9523) | -0.072  (0.5843) | 0.070  (0.5971) |
| REPS1_circ  _0004368 | 0.363  (0.5467) | 0.012  (0.8933) | 0.108  (0.4099) | -0.206  (0.1135) | -0.009  (0.9453) | -0.148  (0.2587) | -0.023  (0.8642) | -0.054  (0.6831) |
| KIDINS220_  circ_0005315 | 4.019  (0.0450) | 0.010  (0.9103) | -0.093  (0.4845) | -0.076  (0.5681) | 0.058  (0.6643) | 0.227  (0.0833) | 0.036  (0.7864) | 0.020  (0.8785) |
| TMEM138_  circ_0002058 | 0.020  (0.8864) | 0.032  (0.7304) | -0.008  (0.9521) | -0.030  (0.8227) | 0.014  (0.9144) | -0.043  (0.7471) | 0.068  (0.6034) | 0.030  (0.8221) |
| MAPK9_circ  _0001566 | 1.954  (0.1621) | 0.044  (0.6357) | -0.119  (0.3663) | 0.063  (0.6301) | 0.062  (0.6389) | 0.202  (0.1208) | 0.057  (0.6662) | -0.010  (0.9406) |
| KIAA1841_  circ_0007793 | 0.034  (0.8546) | -0.014  (0.8824) | -0.179  (0.1715) | -0.027  (0.8372) | 0.089  (0.4983) | 0.025  (0.8473) | 0.050  (0.7058) | 0.244  (0.0601) |
| TMCC1_circ  _0001340 | 0.053  (0.8185) | -0.006  (0.9453) | -0.104  (0.4335) | -0.049  (0.7133) | 0.026  (0.8473) | 0.093  (0.4816) | 0.045  (0.7331) | 0.049  (0.7141) |
| AGTPBP1_  circ_0007162 | 0.068  (0.7941) | 0.033  (0.7198) | -0.047  (0.7215) | -0.076  (0.5616) | 0.168  (0.1995) | 0.156  (0.2330) | 0.110  (0.4023) | 0.264  (0.0413) |
| PSEN1_circ  _0003848 | 0.148  (0.7001) | 0.002  (0.9865) | -0.263  (0.0424) | 0.012  (0.9249) | 0.077  (0.5594) | 0.160  (0.2218) | 0.010  (0.9394) | 0.244  (0.0606) |
| ZNF292_circ  _0004058 | 0.121  (0.7283) | -0.020  (0.8305) | -0.089  (0.5008) | -0.232  (0.0770) | -0.022  (0.8662) | 0.077  (0.5620) | -0.038  (0.7733) | -0.074  (0.5774) |
| FAM120A_  circ_0001875 | 0.047  (0.8279) | -0.082  (0.3747) | -0.237  (0.0687) | -0.145  (0.2675) | -0.070  (0.5942) | 0.129  (0.3253) | -0.041  (0.7551) | -0.009  (0.9473) |
| FUT8_circ  _0003028 | 0.493  (0.4826) | -0.074  (0.4238) | -0.214  (0.1004) | 0.029  (0.8233) | -0.040  (0.7600) | 0.072  (0.5852) | -0.094  (0.4761) | -0.114  (0.3875) |
| KDM4C_circ_0001839 | 0.030  (0.8619) | 0.070  (0.4462) | 0.122  (0.3525) | -0.183  (0.1627) | -0.177  (0.1753) | 0.069  (0.6028) | -0.153  (0.2436) | -0.121  (0.3555) |
| HTT_circ  _0001392 | 1.317  (0.2512) | -0.092  (0.3232) | -0.045  (0.7327) | -0.101  (0.4470) | 0.078  (0.5564) | 0.118  (0.3754) | 0.054  (0.6859) | -0.006  (0.9664) |
| MED12L_circ_0067735 | 1.103  (0.2937) | -0.137  (0.1368) | -0.415  (0.0010) | 0.221  (0.0893) | 0.066  (0.6178) | 0.050  (0.7037) | 0.008  (0.9512) | 0.246  (0.0580) |
| ZFAND6_circ_0000643 | 4.280  (0.0386) | -0.053  (0.5659) | -0.138  (0.2939) | -0.148  (0.2585) | -0.046  (0.7246) | 0.098  (0.4573) | -0.029  (0.8284) | -0.108  (0.4101) |
| SLAIN2_circ  _0126525 | 0.623  (0.4301) | 0.015  (0.8669) | -0.060  (0.6462) | -0.135  (0.3030) | -0.100  (0.4489) | 0.096  (0.4651) | -0.043  (0.7458) | -0.039  (0.7653) |
| PHC3_circ  _0001359 | 0.418  (0.5182) | -0.003  (0.9738) | -0.064  (0.6282) | -0.217  (0.0951) | -0.068  (0.6062) | 0.139  (0.2897) | -0.037  (0.7778) | -0.077  (0.5598) |

Significance level is set at p<0.0005 after Bonferroni correction.
