# Supplementary material for: Differentially Expressed Circular RNAs in Peripheral Blood Mononuclear Cells of Patients with Parkinson's Disease
Source: Mov Disord. 2021 Jan 12;36(5):1170–9. doi: 10.1002/mds.28467 (PMC8248110; doi:10.1002/mds.28467)
Supplement: Supplementary file 7 — Table S5. KEGG and GOslim categories that are mostly deregulated in idiopathic PD. Gene union of the targets of the five miRNAs sequestered by three or more of the deregulated PD circRNAs (miR‐516b‐5p, miR‐526b‐5p, miR‐578, miR‐659‐3p, miR‐1197) in PD versus (A) KEGG and (B) GOslim categories created by the DIANA‐miRPath v3.0 interface using default values (P‐value threshold 0.05, microT‐CDS threshold 0.8). [file MDS-36-1170-s003.docx]

Supplemental Table 5. KEGG and GOslim categories that are mostly deregulated in idiopathic PD. Gene union of the targets of the five miRNAs (miR-516b-5p, miR-526b-5p, miR-578, miR-659-3p, miR-1197) sequestered by half or more of the deregulated PD circRNAs in PD versus (A) KEGG and (B) GOslim categories created by the DIANA-miRPath v3.0 interface using default values (*p*-value threshold 0.05, microT-CDS threshold 0.8).

**A**

| **KEGG pathway** | ***P* value** | **# genes** | **# miRNAs** |
| --- | --- | --- | --- |
| Thyroid hormone signaling pathway | 0.001458 | 24 | 5 |
| Cell cycle | 0.009263 | 25 | 5 |
| Regulation of actin cytoskeleton | 0.014546 | 42 | 5 |
| Rap1 signaling pathway | 0.01546 | 35 | 5 |
| Phosphatidylinositol signaling system | 0.015883 | 16 | 4 |
| MAPK signaling pathway | 0.015883 | 46 | 5 |
| FoxO signaling pathway | 0.015883 | 26 | 5 |

**B**

| **GOslim category** | ***P* value** | **# genes** | **# miRNAs** |
| --- | --- | --- | --- |
| Organelle | 1.13E-78 | 1143 | 5 |
| Cellular nitrogen compound metabolic process | 6.05E-47 | 587 | 5 |
| Cellular protein modification process | 3.93E-19 | 283 | 5 |
| Nucleoplasm | 2.34E-13 | 159 | 5 |
| Enzyme binding | 2.34E-13 | 168 | 5 |
| Protein complex | 4.38E-11 | 411 | 5 |
| Protein binding transcription factor activity | 6.74E-10 | 70 | 5 |
| Cytoskeletal protein binding | 3.91E-09 | 102 | 5 |
| Cell death | 7.91E-08 | 112 | 5 |
| RNA binding | 6.93E-07 | 207 | 5 |
| Nucleic acid binding transcription factor activity | 7.74E-07 | 112 | 5 |
| Macromolecular complex assembly | 3.06E-05 | 95 | 5 |
| Response to stress | 3.72E-05 | 225 | 5 |
| Membrane organization | 4.89E-05 | 65 | 5 |
| Cytoskeleton organization | 0.000178 | 80 | 5 |
